# Vector potential dual effect of promoting the proliferation of chondrocytes and inhibiting the calcification process in the articular cartilage

**DOI:** 10.1038/s41598-023-43949-3

**Published:** 2023-10-06

**Authors:** Hirai Suito, Wataru Minamizono, Nao Yashima, Hiroya Matsunaga, Kaoru Fujikawa, Masafumi Ohsako

**Affiliations:** 1 Graduate School of Human Life Design, Toyo University, 1-7-11 Akabanedai, Kita-Ku, Tokyo, 115-8650 Japan; 2 Japan Society for the Promotion of Science Research Fellowships DC, 5-3-1 Koji-Machi, Chiyoda-Ku, Tokyo, 102-0083 Japan; 3 Graduate School of Health Sports Science, Toyo University, 1-7-11 Akabanedai, Kita-Ku, Tokyo, 115-8650 Japan; 4 Department of Oral Anatomy and Developmental Biology, Showa University School of Density, 1-5-8, Hatanodai, Shinagawa-Ku, Tokyo, Japan

**Keywords:** Cells, Musculoskeletal system

## Abstract

OA commonly affects the articular cartilage of the tibia, and its calcification worsens its advancement and its prevalence has recently increased. Vector potential (VP) represents a novel physical therapy for treating OA. Since the impact of VP on articular cartilage remains unknown, we aimed to assess its effects on articular cartilage and its potential as a new treatment for OA. Here, we divided 24 male Wistar rats, 6-week-old, into control (CO, n = 12) and VP stimulus (n = 12) groups (VP conditions: volt, 67 mV; frequency, 20 kHz; current, 0.12 mA; experimental frequency, 30 min/days, 5 days/week, and 3 weeks). Articular cartilage can be classified into four layers: superficial, medial, deep, and calcified. Moreover, the number of chondrocytes in the articular cartilage was higher in the CO group compared to the VP group, although the calcified layer was thinner in the VP group. Furthermore, *MKi67* exhibited higher expression in the VP group than in the CO group, while ectonucleotide pyrophosphatase/phosphodiesterase 1 was downregulated in the VP group. Our findings indicate that VP positively influenced chondrocyte proliferation and inhibited calcification in articular cartilage. Thus, VP stimulation may assist in the development of novel strategies for preventing OA.

## Introduction

Osteoarthritis (OA) primarily affects the articular cartilage and is caused by various factors, including aging, trauma, and obesity^1–3^. Patients with OA typically exhibit thinner articular cartilage compared to those without this condition^4,5^. While drugs and physical therapy have been employed as preventive measures^6,7^, these strategies primarily focus on slowing OA progression rather than treating post-articular cartilage lesions. This is likely due to the unique histological structure of articular cartilage, which lacks innervations and vascular structures^8^. Angiogenesis plays a vital role in tissue repair^9^, requiring the presence of blood vessels and nerves. However, articular cartilage regeneration is often limited due to OA-induced calcification^10^. This process is normally regulated by the interplay between ectonucleotide pyrophosphatase/phosphodiesterase 1 (ENPP1, known to suppress calcification) and tissue non-specific alkaline phosphatase (TNAP, known to promote calcification). OA occurs as a result of ENPP1 downregulation and TNAP upregulation^11,12^, making it important to maintain the articular cartilage thickness to prevent OA and delay calcification. A prior study suggested that preventive measures against OA could also be utilized for its treatment, mainly because OA decreases cancellous bone volume^13^. For instance, bisphosphonates, commonly used in the treatment of osteoporosis, have been explored as treatments for OA due to their ability to combat the synthesis of inflammatory cytokines and preserve articular cartilage^14^. Similarly, anti-inflammatory drugs, such as NSAIDs and opioids, have also been employed to treat OA^15^. However, these drugs may adversely affect tissue structure in vivo. In contrast, physical stimulation (e.g., pulsed electromagnetic stimulation) showed little or no improvement of OA^16^. Therefore, alternative treatment strategies need to be developed.

Recently, a vector potential (VP) generator was developed, characterized by being energized without requiring a stimulus. Moreover, it can be stimulated without direct contact with the body. Meanwhile, transcutaneous electric nerve stimulation (TENS) is commonly used to treat OA^17^, although its precise mechanism of action remains unclear. Recent studies have reported that TENS stimulates ions inside and outside the cells to increase cellular activity^18^. In addition, TENS operates by generating electric fields, which requires the presence of a VP^19^. Hence, we hypothesized that VP also influences the structure of cartilage tissue. Moreover, the proliferation of chondrocytes in articular cartilage leads to the synthesis of a protein called “Ki67”^20^. Generally, cell proliferation occurs in four phases: G1, S, G2, and M^21^, with the non-proliferative phase referred to as the G0 phase^21^. Recently, Bakuchiol, a Chinese herbal medicine, has been found to induce articular chondrocyte proliferation, potentially offering treatment options for OA.

The extracellular matrix and chondrocytes play a critical role in protecting articular cartilage. The extracellular matrix consists of collagen fibers and an amorphous substrate, central to articular protection. Type II collagen (Col2) is the primary component of cartilage^22^, while the amorphous substrate primarily comprises aggrecan^23^, well-known for its water-retaining properties. Aggrecan also binds to Col2, and the synthesis of both components is essential for maintaining articular cartilage health^24^. In OA, the disrupted balance in cartilage tissue leads to reduced synthesis of Col2 and aggrecan^25^. Therefore, the secretion of Col2 and aggrecan plays a protective role in preventing OA in articular cartilage.

**Figure 1:**
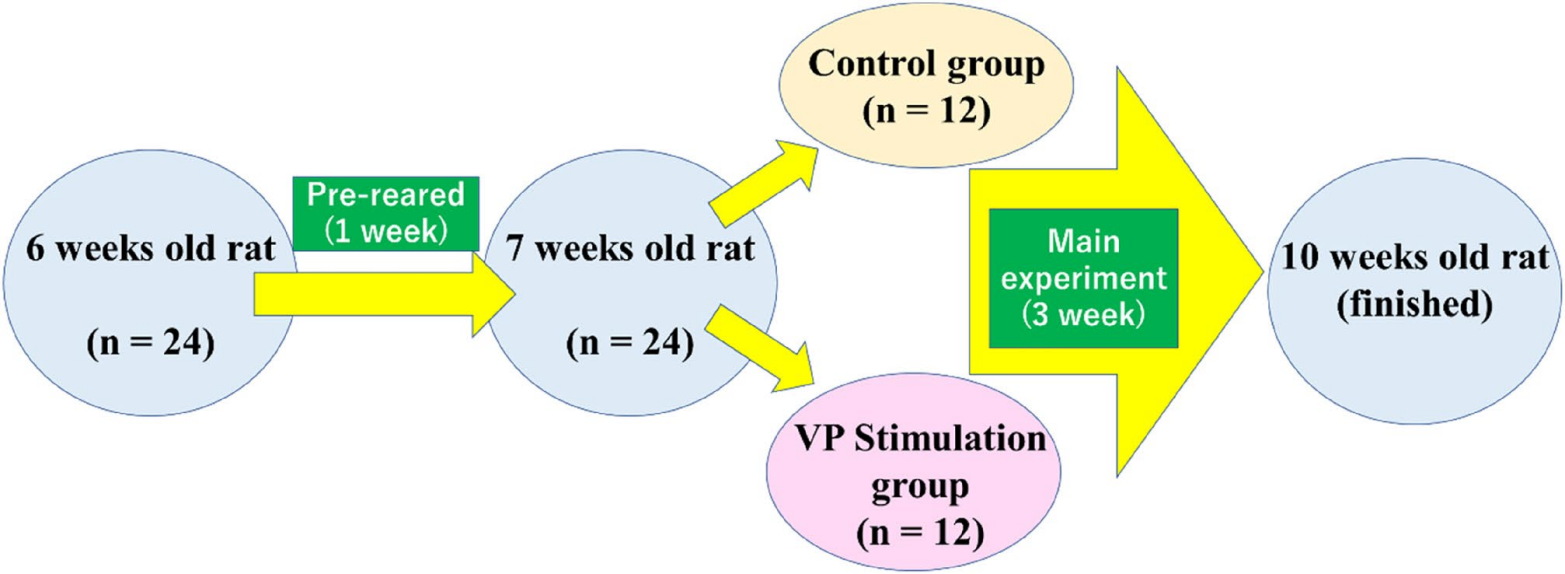
Experimental design.

As mentioned earlier, the ability to synthesize Col2 and aggrecan is diminished in the articular cartilage of patients with OA, thereby limiting calcification. Despite previous studies emphasizing the importance of improving cartilage tissue function to prevent OA, scientific evidence regarding the effects of VPs on articular cartilage remains scarce. Therefore, this study aimed to provide histological and biochemical evidence regarding the impact of VP on articular cartilage (Fig. 1).

## Results

### Calcification in articular cartilage is inhibited by the VP

Histological examinations of the articular cartilage revealed distinctive characteristics in each layer. In the superficial layer, flat chondrocytes were observed, while the middle layer exhibited metachromasia, as demonstrated by toluidine blue staining, revealing a high concentration of proteoglycans. In contrast, the deep layer did not exhibit metachromasia due to its abundance of collagen fibers. Finally, the calcification layer showed a distinct staining pattern compared to the other layers, indicating its unique composition and properties.

**Figure 2:**
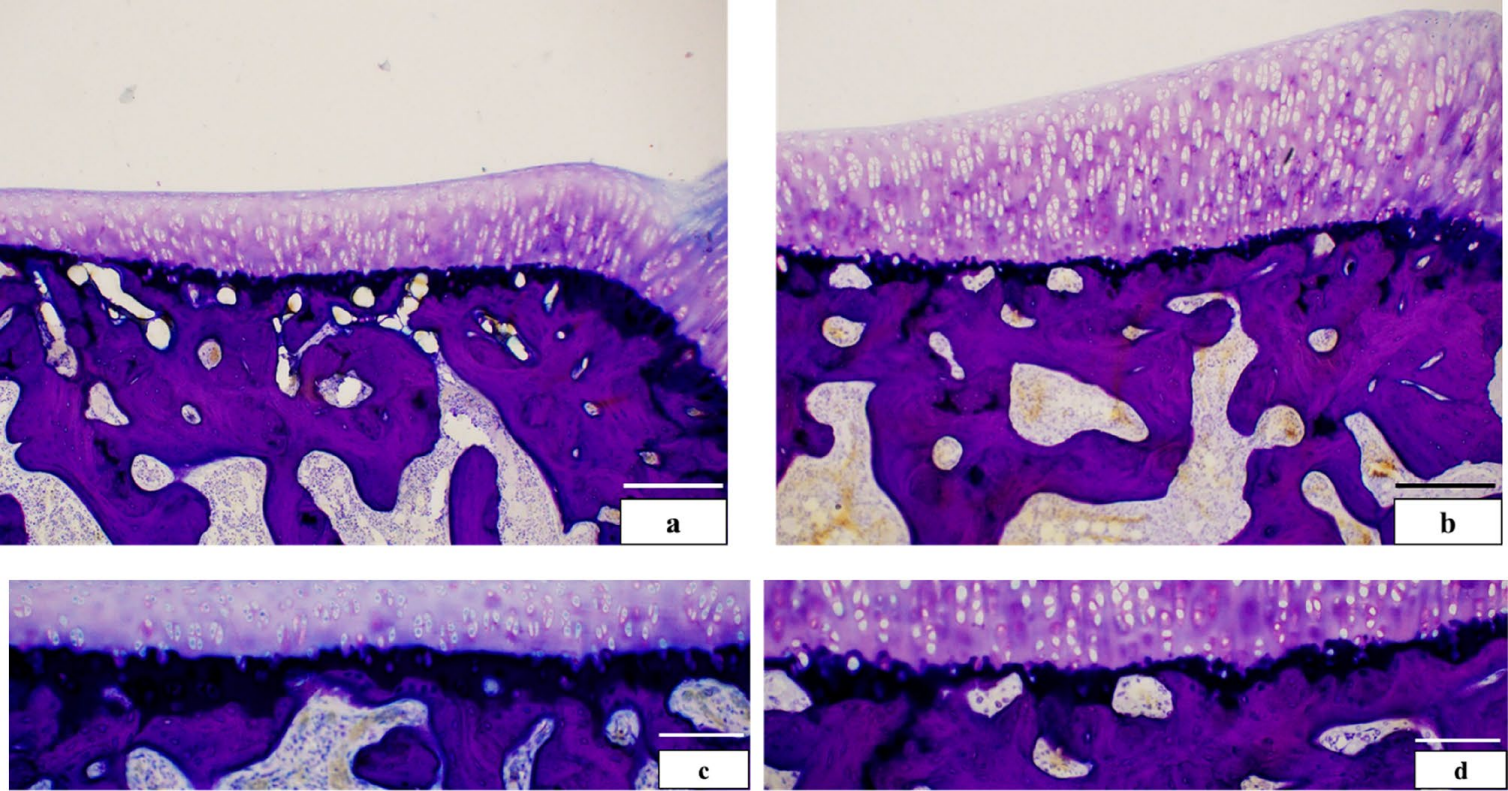
Comparison of basic structures in articular cartilage (**a**,**b** bar = 200 µm, **c**,**d**: = 100 µm, non-decalcification specimen in toluidine blue staining). The articular cartilage in the CO (**a**) was thinner than that in the VP (**b**), and the calcification layer in the CO was thinner than that in the VP. Comparison of the calcification layer, CO (**c**) was thicker than VP (**d**). *S* superficial layer, *M* medial layer, *D* deep layer, *C* calcification layer.

**Figure 3:**
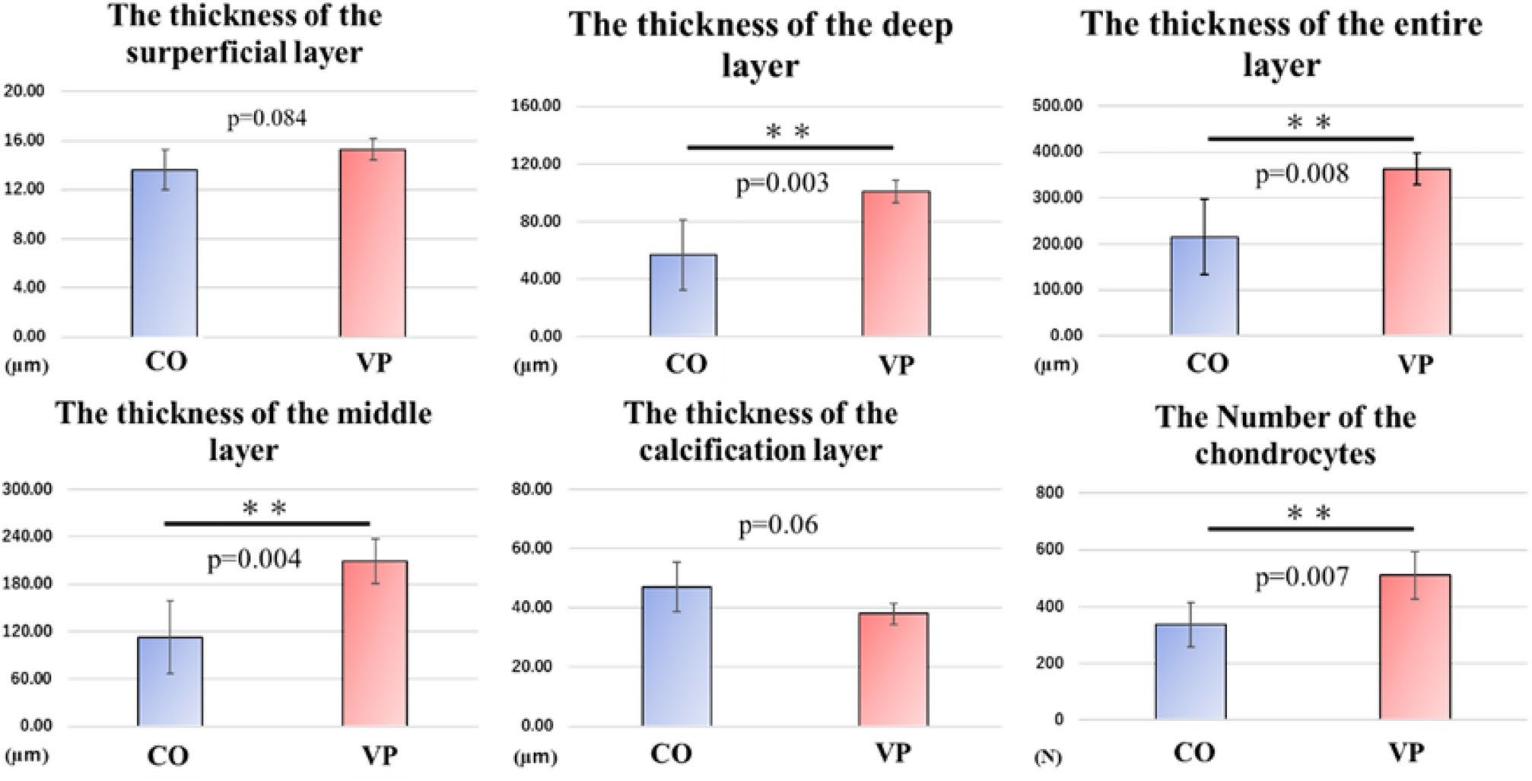
Morphometry in the articular cartilage (Rigorac resin specimen in toluidine blue staining, n = 6). Compared to the CO group, the middle (P = 0.004) and deep layers (P = 0.003) thicknesses were significantly greater in the VP group. The thicknesses of the superficial (P = 0.084) and calcified (P = 0.06) layers were not significantly correlated; however, the thickness of the calcified layer in the VP exhibited a decreasing trend. Furthermore, despite these results, significantly higher thickness values of the entire articular cartilage were observed in the VP (P = 0.008). In addition, the number of chondrocytes in the VP group was significantly higher than that in the CO group (P = 0.007).

**Figure 4:**
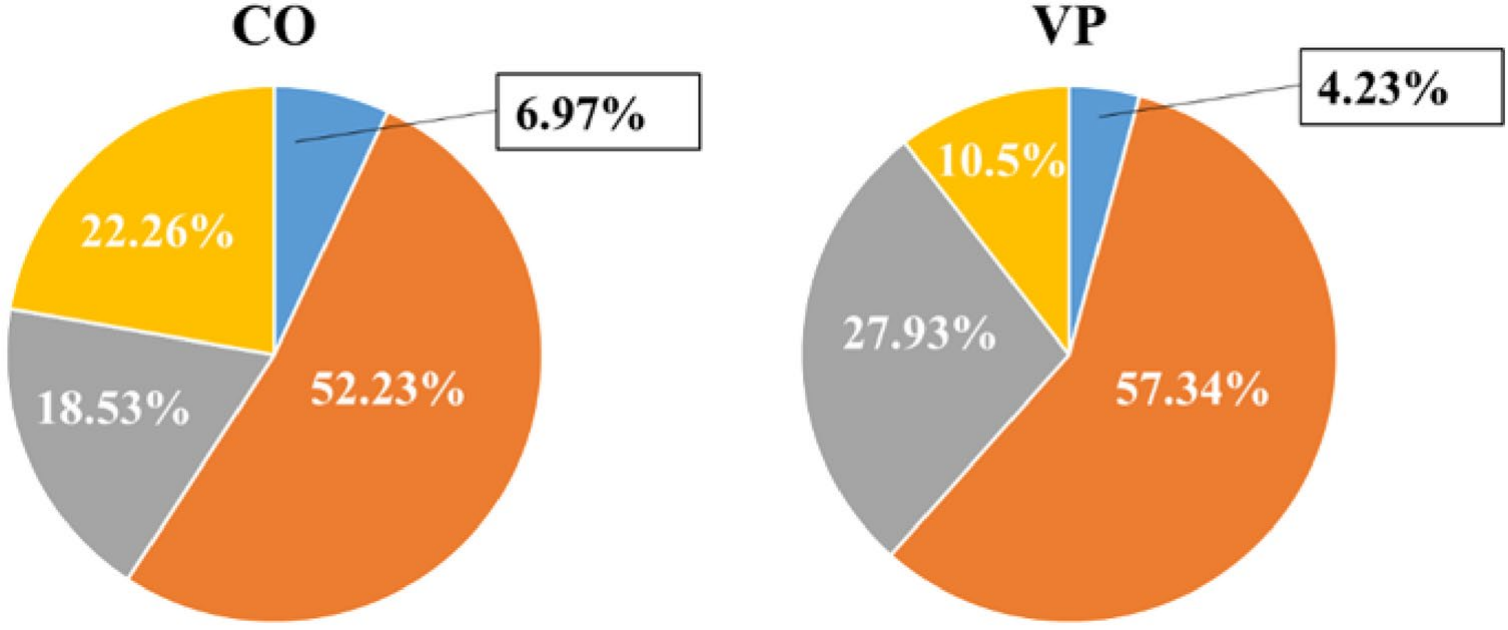
Rate of the articular cartilage in each layer. The rates in the superficial and calcification layers did not mostly differ changed. The deep layer in the VP group had a higher score than that in the CO group; however, the calcification layer had a lower score than that in the CO group.

The articular cartilage in both groups was histologically divided into four layers: superficial, middle, deep, and calcified (Fig. 2a,b). The observed thickness of the calcification layer in the VP group was thinner than that in the CO group (Fig. 2c,d), prompting further investigation through histomorphological analysis. While the thickness of the superficial (P = 0.084) and calcification (P = 0.06) layers did not significantly differ between both groups, the middle (P = 0.004), deep (P = 0.003), and entire layers (P = 0.008) were significantly thicker in the VP group compared to the CO group (Fig. 3). Consequently, we focused on the articular cartilage formation rate in each layer (Fig. 4) revealing that the formation rates of the superficial and middle layers did not differ significantly. However, the rate of the deep layer in the CO group was 18.53%, whereas in the VP group, it was 27.93%. Additionally, the calcification rate in the CO and VP groups was 22.26 and 10.5%, respectively. These findings suggest that the overall thickening of the articular cartilage in the VP, despite the thinning of the calcified layer, is likely due to the thickening of the middle and deep layers.

### Articular cartilage thickened by VP

**Figure 5:**
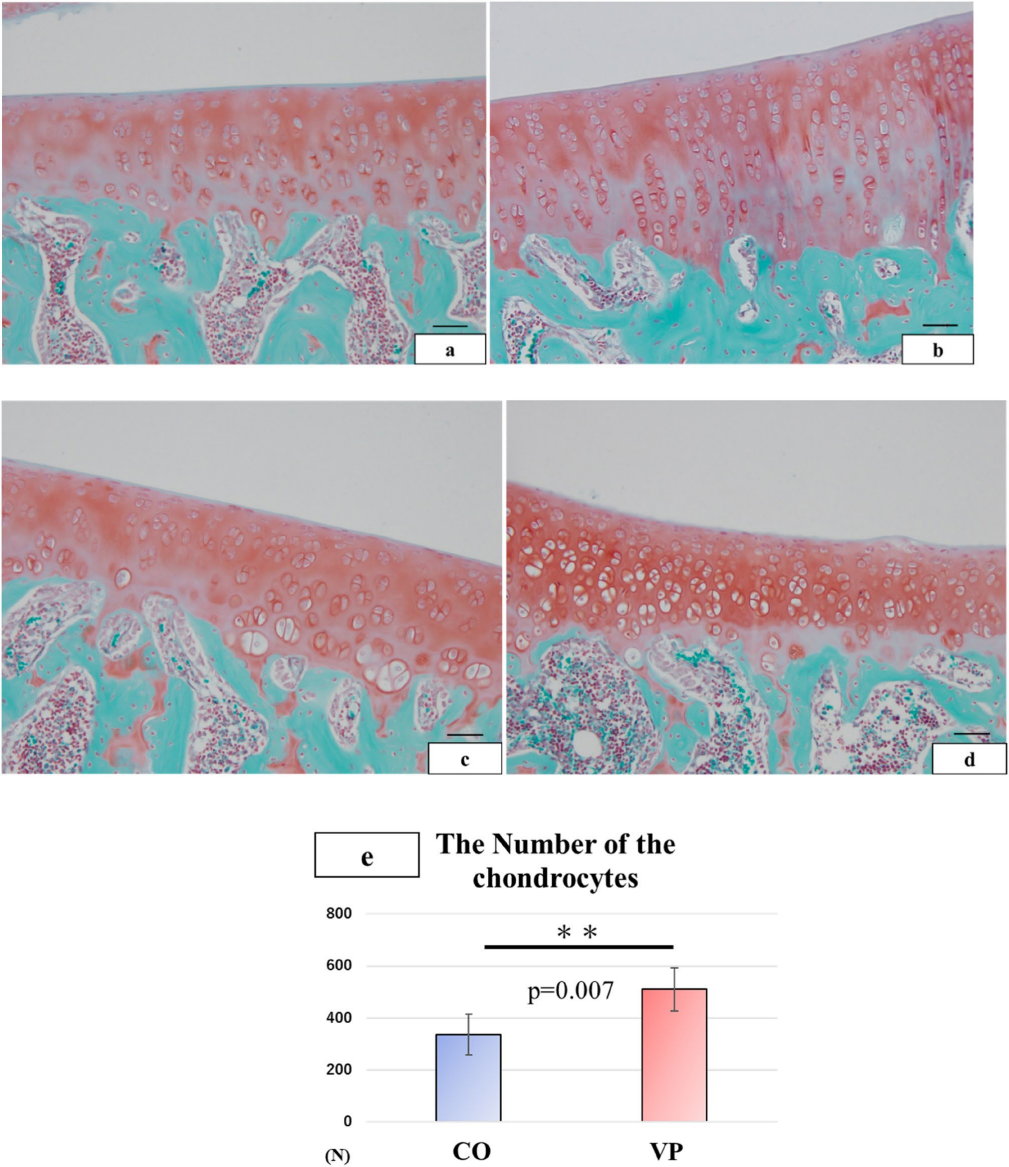
Morphological structure of the articular cartilage and the number of chondrocytes (**a**–**d** bar = 50 µm, embedding-paraffin specimen). The medial articular cartilage in CO (**a**) is thinner than that in VP (**b**). The lateral articular cartilage in the CO (**c**) was also lower than that in the VP. (**d**) The number of chondrocytes in the VP was higher than that in the CO (P = 0.007).

In both groups, the middle layer displayed a high concentration of chondrocytes. Specifically, in the middle layer of both the medial (Fig. 5a,b) and lateral (Fig. 5c,d) sides, the VP group exhibited a greater number of chondrocytes compared to the CO group. Chondrocytes in the articular cartilage of the VP group were more densely distributed and had higher counts than those in the CO group. Notably, chondrocytes in the VP group formed a longitudinal arrangement. Furthermore, the number of chondrocytes in the VP group exceeded that in the CO group (P = 0.007) (Fig. 5e). As a result, these findings suggest that the increase in articular cartilage thickness attributable to VP is linked to the heightened chondrocyte population.

### Higher chondrocyte activity and calcification suppression induced by VP

**Figure 6:**
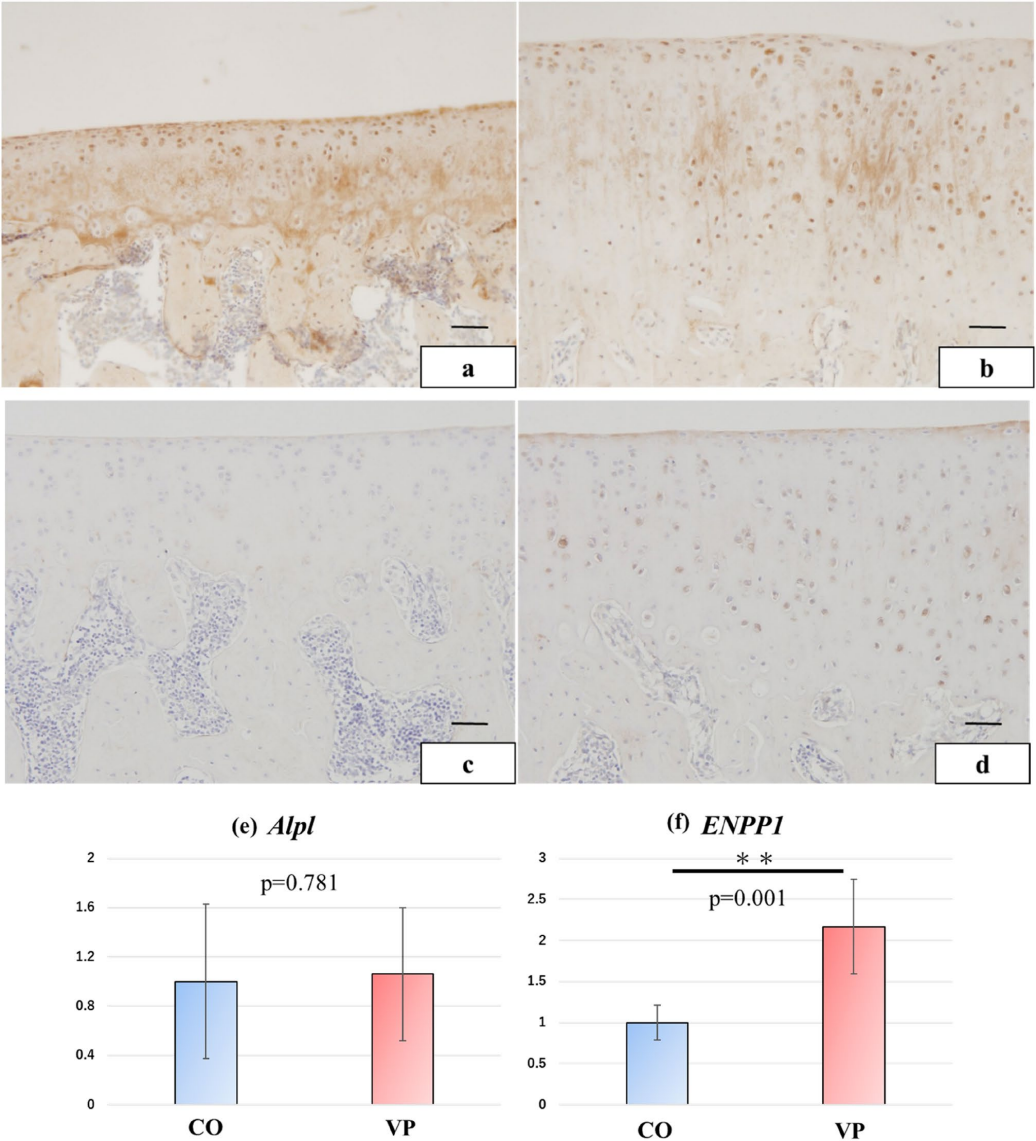
The condition of calcification factor (**a**–**d** embedding-paraffin specimen, **e**,**f** qPCR analysis). Immunolocalization of TNAP in the CO was observed in the superficial and deep layers (**a**); however, the VP reacted in the middle layer (**b**). The immunoreactivity of ENPP1 in CO was not observed in the articular cartilage (**c**), but VP was confirmed in positive cells (**d**). The expression of *Alpl,* does not change the score between CO and VP (**e**). However, *Enpp1* expression in VP was significantly higher than that in CO (**f**).

Immunolocalization of TNAP, known as the calcification-promoting factor, was prominently observed in the superficial and deep layers of the matrix and chondrocytes in the CO group (Fig. 6a). In contrast, TNAP immunoreactivity in the VP group was weakly observed in the middle layer (Fig. 6b). Moreover, CO mostly did not exhibit a reaction in the superficial layer or among the chondrocytes (Fig. 6c), whereas ENPP1 immunoreactivity in the VP group was observed from the surface to the deep layer within the chondrocytes (Fig. 6d). Furthermore, *Enpp1* expression was significantly higher in the VP group compared to the CO group (P = 0.001), while *Alpl*, which encodes tissue non-specific alkaline phosphatase, expression did not show a significant difference (P = 0.781) (Fig. 6e,f). Thus, these findings indicate that VP suppressed calcification by downregulating TNAP synthesis and upregulating *Enpp1*.

**Figure 7:**
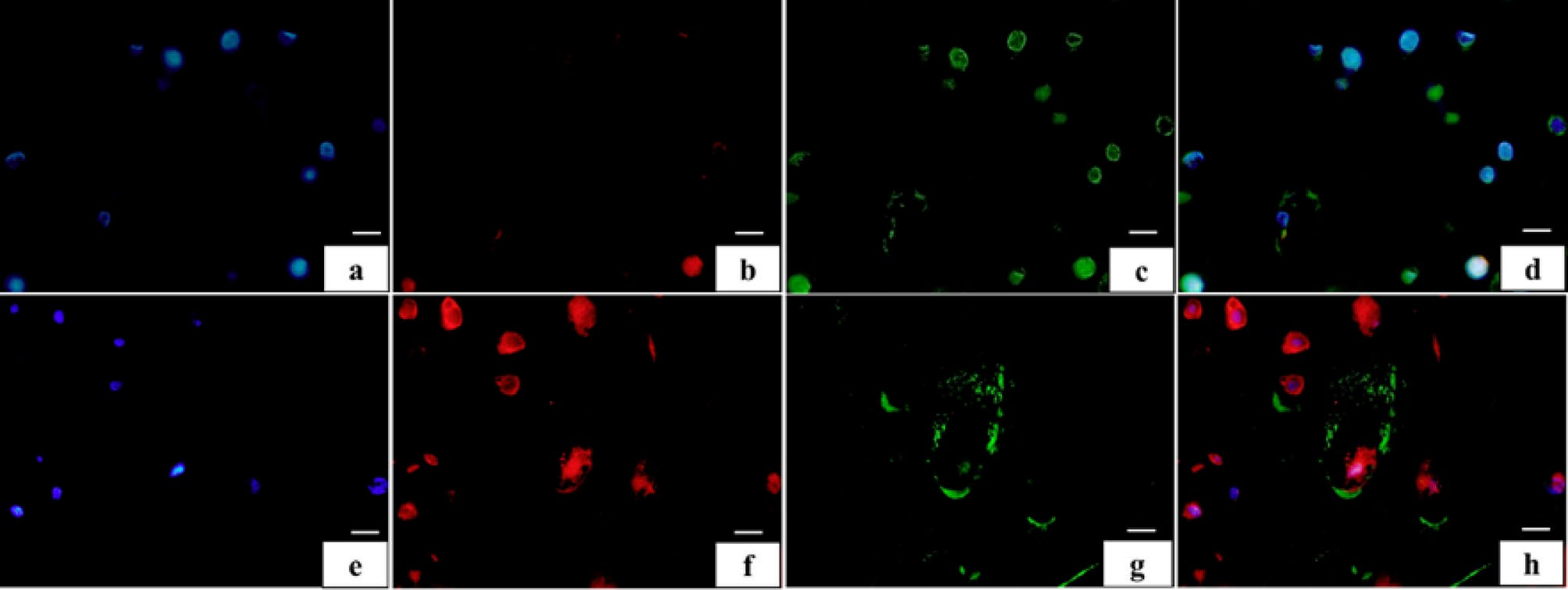
Synthesis condition of collagen in the deep layer (**a**–**d** CO, **e**–**h** VP, **a**,**e** DAPI, **b**,**f** Col2, **c**,**g** Col10, and **d**,**h** merge image). DAPI-positive cells were observed in CO (**a**) and VP (**e**). However, col2-positive cells in CO (**b**) were not observed in the deep layer, whereas in VP (**f**) they were observed in the deep layer. In contrast, Col10 reacted in several layers in the CO (**c**); however, in VP (**g**), Col10 was not observed in the deep layers.

DAPI-positive cells were observed in both groups (Fig. 7a,e). Col2-positive cells in the deep layer of CO group were mostly absent; however, the VP group showed an increase in the number of positive cells compared to the CO group (Fig. 7b,f). Meanwhile, Col10 immunolocalization of the deep layer of the CO group was detected in cartilage lacunae and chondrocytes (Fig. 7c). Similarly, the VP group also displayed Col10 immunoreactivity in the cartilage lacuna, although it was lower compared to what was observed in the CO group (Fig. 7g). The overlay image of the CO group strongly revealed Col10; however, the VP’s group overlay image revealed not only Col10 but also Col2 positive reactions (Fig. 7d,h).

### Chondrocyte proliferation promoted by VP

**Figure 8:**
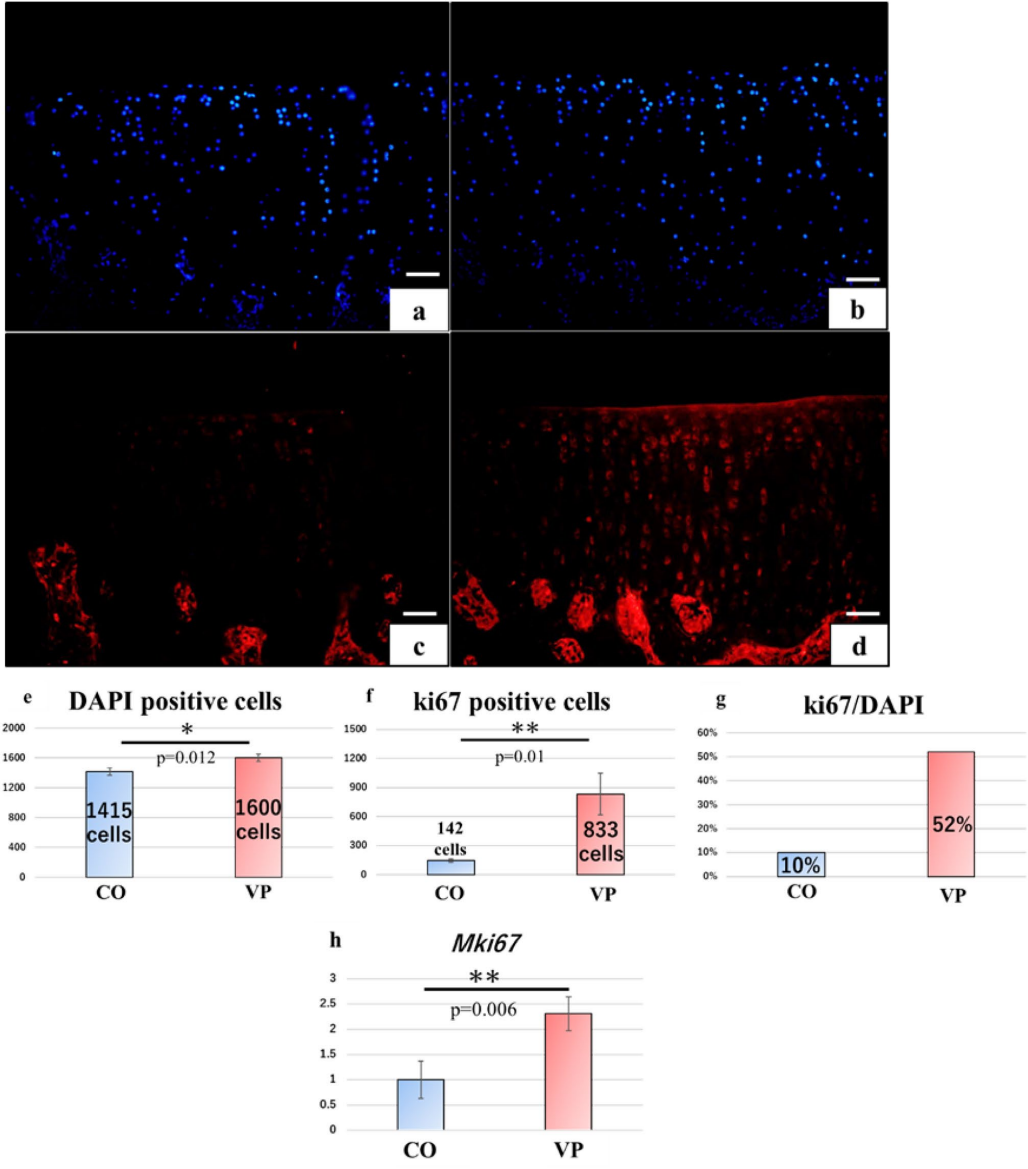
The observation focused on the proliferation of chondrocytes in the articular cartilage (**a**,**c** CO, **b**,**d** VP, **a**,**b** DAPI, **c**,**d** ki67, **e**–**g** the number of the positive cell, and **h** qPCR analysis; however, measurement of positive cells was performed with n = 3). DAPI-(**a**,**b**) and ki67-positive cells (**c**,**d**) were observed in both groups. The number of DAPI and ki67-positive cells in the VP was significantly higher than that in the CO (**e**,**f**). The rate of ki67/DAPI in the VP was higher than that in the CO (**g**). The expression of *Mki67* in VP was significantly higher than that in CO (**h**).

The number of 4',6-diamidino-2phenylindole (DAPI)-positive cells was higher in the articular cartilage of the VP group compared to the CO group (Fig. 8a,b). Ki67-positive cells were distributed throughout the layers of the VP group; however, their reaction in the CO group was notably weak (Fig. 8c,d). In line with these findings, the number of DAPI-positive cells (P = 0.012) and ki67-positive cells (P = 0.01) in the VP group was significantly higher than that in the CO group (Fig. 8e,f). Notably, the percentage of ki67-positive cells was observed to be higher in the VP group (Fig. 8g), and *Mki67*, which encodes ki67, exhibited a significant increase in expression in the VP group (P = 0.006) (Fig. 8h). Consequently, these findings suggest that VP not only enhanced chondrocyte proliferation but also preserved the proliferative function of chondrocytes.

## Discussion

At the structural level, articular cartilage is typically divided into four layers: the superficial layer, middle layer, deep layer, and calcification layer^27^. The boundary between the deep and calcification layers is known as the “tidemark,” and its elevation is a characteristic feature of OA^28^. Once articular cartilage undergoes degeneration, it does not naturally regenerate over the individual’s lifespan. Thus, maintaining the thickness and tidemark of the articular cartilage is likely the most straightforward way to prevent OA. Our study revealed that the overall thickness of the articular cartilage was higher in the VP group than in the CO group, despite a thinner calcification layer. This suggests that VP may be effective in preventing OA. Additionally, the middle layer of articular cartilage secretes a cartilage matrix and contains highly active chondrocytes^27^. Therefore, chondrocytes in the middle layer play a crucial role in determining the thickness of the articular cartilage, contributing to its structural maintenance.

In this study, VP activity was detected in various types of chondrocytes. Particularly, the articular cartilage in the VP group exhibited thickening, and the proportion of the middle layer was notably high. These findings indicate that the middle layer of the VP thickens articular cartilage by increasing the number of chondrocytes. In contrast, Col2 is predominantly localized in the superficial layer^29^, while Col10 serves as a marker for the calcified cartilage matrix^30^. Typically, immature chondrocytes synthesize a non-calcified cartilage matrix^31^. As differentiation progresses, chondrocytes hypertrophy, and the matrix surrounding them becomes calcified^32^. Moreover, immature chondrocytes are found predominantly in areas where Col2 is present, while hypertrophic chondrocytes are primarily located where Col10 is expressed, as Col2 is synthesized in less-differentiated chondrocytes and Col10 is synthesized in more-differentiated chondrocytes. Interestingly, Col10 was detected in the deep layers of both groups; however, the deep layer of the VP group not only exhibited Col10 but also a significant number of Col2 reactions. This suggests that chondrocytes in the articular cartilage of the VP group may experience suppression during the differentiation process. Furthermore, chondrocytes resembling those in the middle layer were also observed in the deep layer of the articular cartilage. However, since chondrocyte differentiation factors were not specifically identified in this study, further investigation is necessary to reach a more definitive conclusion.

In vivo, tissues are regulated by interactions between ENPP1 and TNAP. ENPP1 functions to suppress calcification^33^, while TNAP promotes calcification^34^. This interaction hinges on the formation of hydroxyapatite, a substance involved in various types of tissue calcification, including hard tissue calcification and ectopic calcification^35,36^. However, the formation of hydroxyapatite is inhibited by pyrophosphoric acid. Pyrophosphoric acid is mainly synthesized by ENPP1^37^ and is degraded by TNAP into inorganic phosphate^38^. In contrast to pyrophosphoric acid, inorganic phosphates facilitate the deposition of hydroxyapatite^37^. Consequently, downregulation of ENPP1 and/or upregulation of TNAP promote calcification in vivo. Notably, in OA, the articular cartilage not only undergoes calcification but also experiences downregulation of ENPP1 and upregulation of TNAP^38,39^. Therefore, delaying the calcification of articular cartilage could potentially prevent OA. However, excessive delay in calcification may lead to pathological changes in articular cartilage since the calcified layer of articular cartilage is vital for connecting it to the subchondral bone^40^. Although this study did not reveal a significant difference in the thickness of the calcification, immunostaining and gene expression indicated a slower progression of calcification in the VP group. These findings suggest that while VP may suppress articular cartilage calcification, it is unlikely to induce a pathological state, as the process occurs gradually rather than excessively. Additionally, the calcification layers in the VP group were thinner than those in the CO group, despite showing similar histomorphometric results. Furthermore, we observed strong TNAP reactivity and weak ENPP1 reactivity in the CO group, while the VP group exhibited contrasting results. This suggests that the VP stimulus may delay calcification in articular cartilage. The gene expression findings in this study are equally significant. *Enpp1* expression in the VP group was significantly upregulated compared to that in the CO group, whereas *Alpl* expression showed no notable change. Therefore, it is reasonable to assume that the VP stimulus activates ENPP1 and the synthesis of TNAP without affecting the gene expression of *Alpl*. Moreover, the articular cartilage in the VP group exhibited delays in the onset of OA, suggesting a reduced incidence rate of this condition. Consequently, VP delays calcification by activating ENPP1. To further explore the calcification aspect, our focus was directed toward studying the immunolocalization of TNAP. TNAP immunolocalization in the CO group was observed predominantly in the matrix of the surface and deep layers. In contrast, TNAP immunolocalization in the VP group was weakly observed in the middle layers. Based on this, it is known that TNAP immunolocalization, a crucial factor for promoting calcification in articular cartilage, is speculated to be associated with the calcified region. Thus, in articular cartilage, TNAP may serve as an indicator of calcification in the deeper layers above the calcified layer. Moreover, the stimulation of the VP is thought to activate not only ENPP1 but also influence TNAP immunolocalization in the calcification of the articular cartilage.

Cell proliferation typically occurs in four cycles: G1, S, G2, and M. During the G1 phase, DNA synthesis and replication are preserved, and subsequently, S-phase cells progress to the G2 phase to maintain cell division. Following this, cells divide and proliferate during the M phase, and the cell cycle is once again dislocated from M to G1 phase. Ki67 is specifically synthesized from the G1 phase into M and is widely employed for cell observation^41^. Therefore, *MKi67* expression represent a proliferative process. In this study, the number of Ki67-positive cells in the VP group was significantly higher than that in the CO, with DAPI-positive cells showing a similar trend. Moreover, the rate of Ki67 positive reaction in the CO group was only 10%, while in the VP group, it reached 52%. Furthermore, *MKi67*expression was significantly higher in the VP group than in the CO group. These findings indicate that VP irradiation promotes the proliferation of articular chondrocytes. Moreover, based on the *MKi67* results, the potential proliferative capacity of articular cartilage also increased, suggesting that chondrocytes continued to proliferate for some time following VP irradiation.

This study has some limitations. First, it primarily focused on assessing the influence of VP stimulation on the structure of articular cartilage, and it does not delve into its effects on other skeletal tissues, such as bone, muscle, tendon, and ligament. Future studies should consider exploring the impact of VP on these tissues. Secondly, the sensitivity of chondrocytes to VP stimulation was not comprehensively examined in this study. Understanding the precise mechanisms and specific pathways involved in the response of chondrocytes to VP stimulation would provide valuable insights.

In conclusion, our study has demonstrated that VP stimulation enhances the proliferation of articular chondrocytes and retards the calcification of articular cartilage. Additionally, VP stimulation activates the function of articular cartilage. Hence, based on these findings, we posit that VP stimulus may aid in the development of innovative strategies for preventing OA. Although the methodology employed in this study has enabled us to comprehend the effects on the structure of articular cartilage, the optimal treatment regimen remains uncertain. Therefore, we intend to analyze the effects of different VP irradiation conditions on articular cartilage to determine the most effective conditions.

## Methods

### ARRIVE guidelines

This study was performed in accordance with Essential 10 in the ARRIVE 2.0 guidelines. The details of each item are provided below. We also confirmed that all experiments were conducted in accordance with the pertinent guidelines and regulations.

### Animals

A total of 24 male rats, aged 6 weeks (Wistar Nippon Bio-Sup. Center, Tokyo, Japan) were used in this study. All rats were housed under specific pathogen-free conditions and were pre-conditioned for 1 week to acclimatize to their new environment. Subsequently, they were randomly assigned to either the VP stimulus group (n = 12) or the control group (CO, n = 12). The housing facilities were maintained in a consistently clean environment, with up to four rats per cage and continuous access to water and food ad libitum (Oriental Yeast Co. LTD, Tokyo, Japan). The experiments were conducted at the Asaka Campus of Toyo University, situated approximately 7 m above sea level. Ethical approval for the experiments was obtained from the Committee of Animal Experiments and Ethics for Research, Graduate School of Human Life Design, Toyo University (Tokyo, Japan; Approval No.2022-36.).

### Experimental design

Firstly, stringent hygiene protocols were performed throughout the study. Prior to entering the breeding area and during the experiments, hand disinfection was performed. Latex gloves were worn during the experiments. Moreover, cage sanitation was diligently maintained to minimize rik of disease transmission.

VP was systemically stimulated to the rats under anesthesia using a VP generator (Sumida Electric Co., Ltd.). The VP parameters were as follows: volt, 60 mV; frequency, 20 kHz; current, 0.12 mA; experimental frequency, 30 min/days, 5 days/week, for a total of 3 weeks. After the experimental period, all rats were euthanized using CO_2_ gas. The right legs were employed as specimens for histological analysis, while the left legs were utilized for gene expression analyses and histomorphometry. Since the relationship between VP and electric field strength can be expressed using a mathematical formula, allowing the calculation of VP strength from electric field strength, and vice versa. In this study, the electric field within the device was measured to be 0.22 V/m (calculated by dividing the voltage, 60 mV). Notably, this electric field strength matched the value used during the device design, affirming the generation of VP within the device. Additionally, the VP generation did not result in an increase in temperature. Importantly, this experiment was consistently conducted at 10:00 a.m. to minimize the influence of circadian rhythms during VP stimulation. To expedite the time between exposure to CO_2_ and loss of consciousness, the CO_2_ concentration was gradually increased at a displacement rate of 30–70%/min of container’s volume. Following respiratory arrest, the subjects were exposed to CO_2_ gas for a minimum of 1 min.

### Inclusion and exclusion criteria and randomization

All the rats used in this study were included in the analysis. The right limbs of all rats were used for histological analysis (n = 12), whereas the left limbs were used for bone morphometry and gene expression analyses (n = 6). The assignment of animals to the experimental groups was conducted through a randomized process involving the drawing of cards from a shuffled set of numbers ranging from 1 to 24. There were 12 assignment cards for each group, and the rats were allocated to their respective groups based on the numbers drawn. The experimental procedures and initial assessments of the results were carried out by H.S. Subsequently, these results were shared with five blind operators (K.F., W.M., N.Y., H.M., and M.O.) to perform the pertinent analyses.

### Non-decalcified specimens

The non-decalcified specimens were subjected to a staining process using a 1% toluidine blue solution for a duration of 45 s. This staining was carried out after embedding the specimens in Rigolac resin (3801TB12T; NISSHIN EM, Tokyo, Japan) to facilitate the visualization of calcification within the tibial articular cartilage. Furthermore, these specimens served as the basis for histological and morphological examinations.

### Morphological analyses

Morphological analyses (n = 6) were conducted on six parameters: the thickness of the superficial, middle, deep, calcification, and entire layers, as well as the chondrocyte count. The measurements for all thickness parameters was based on histological observations. For instance, the superficial layer of articular cartilage was characterized by the presence of flat chondrocytes, while the middle layer exhibited metachromasia due to a high concentration of proteoglycans. In contrast, the deep layer displayed a limited metachromatic reaction, indicating lower proteoglycan content, and the calcification layer exhibited a distinct dark violet coloration following toluidine blue staining. Additionally, the chondrocyte count in the articular cartilage encompassed the totality of chondrocytes present. Furthermore, all morphological analyses were performed blindly on both groups, outsourced for impartiality.

### Decalcification embedding-paraffin sections

The specimens were first decalcified using an 8% ethylenediaminetetraacetic acid solution (349-01863; Dojindo, Kumamoto, Japan) over a 3-week period. Subsequently, they were embedded in paraffin (415-25791; FUJIFILM Wako Pure Chemical Corporation) and sliced into 4 µm-thick sections. The decalcified specimens were sectioned using a REM-710 Retoratome (Yamato, Saitama, Japan) and observed under a light microscope (BX53F; OLYMPUS, Tokyo, Japan) following safranin O staining for 5 min. Immunohistochemistry was also performed. All specimen observation sites were centrally designated, and precise thin slices were prepared while monitoring the structural integrity of the growth plate.

### Immunohistochemistry

Initially, the paraffin sections were removed using xylene after warming for 1 h in a wet box. Subsequently, enzymatic digestion with hyaluronidase (18240-36; Nacalai Tesque, Inc., Kyoto, Japan) was performed to expose the antigens in the specimens. Additionally, to minimize non-specific reactions, the specimens were blocked using 3% bovine serum albumin. Then, the specimens were subjected to incubation with the following primary antibodies at 4 °C: anti-ki67 (× 200, M7240, Abcam, Cambridge, United Kingdom), anti-TNAP (× 300, ab65834, Genetex), anti-ENPP1 (× 300, rabbit, Genetex), anti-Col2 (× 300, MA5-12789, Thermo Fisher, Tokyo, Japan), and anti-Col10 (× 300, GTX37732, Genetex). Finally, the specimens were further incubated with secondary antibodies for 30 min at 25 °C and then mounted with a DAPI-containing mountant for fluorescence observation (Abcam). The histological analysis, specifically the counting of Ki67-positive cells, was performed on samples from three rats, with all positive cells counted through visual inspection. Furthermore, standardized exposure times of 250 ms for Ki67 and 3 ms for DAPI were applied, and the enumeration of positive cells was performed visually. For light observations, TNAP and ENPP1 were combined with diaminobenzidine.

### Quantitative reverse transcription-polymerase chain reaction (qRT-PCR)

Gene expression levels within the tibial tuberosity were calculated using the CFX96 Real-Time System (Bio-Rad, Hercules, CA, USA). Total RNA was extracted from the tibial articular cartilage using TRIzol (15596026; Thermo Fisher Scientific, Chiba, Japan). To ensure RNA purity (with a 260/280 ratio between 1.8 and 2.0) tRNA was analyzed using NanoDrop One (Termo Fisher). Subsequently, cDNA was synthesized using the iScript gDNA Clear synthesis kit (172530; Bio-Rad Laboratories, Hercules, CA, USA). The concentration of tRNA during cDNA synthesis was standardized to 10 ng/µl. Gene expression analysis was performed using the TaqMan probe assay. Furthermore, 45S ribosomal RNA was used as a housekeeping gene. The PCR conditions were as follows: initial denaturation at 95 °C for 20 s, then 40 cycles of denaturation at 95 °C for 15 s, and a final extension at 60 °C for 1 min. *Rn45S* mRNA was used as an internal control. The relative expression levels were calculated using the 2^−ΔΔCT^ method^26^.

### Statistical analysis

All statistical analyses were performed using the SPSS ver.26 (SPSS Inc., Chicago, IL, USA). A *t*-test analysis was performed to determine the correlation between the two groups. A P-value < 0.05 was considered to be statistically significant.

## Data Availability

The data that support the findings of this study are available in this manuscript.
